# Antimicrobial stewardship for sepsis in the intensive care unit: Survey of critical care and infectious diseases physicians

**DOI:** 10.1017/ice.2021.389

**Published:** 2022-10

**Authors:** M. Cristina Vazquez Guillamet, Jason P. Burnham, Maria Pérez, Marin H. Kollef, Constantine A. Manthous, Donna B. Jeffe

**Affiliations:** 1 Division of Infectious Diseases, Washington University School of Medicine, St Louis, Missouri; 2 Division of Pulmonary and Critical Care Medicine, Washington University School of Medicine, St Louis, Missouri; 3 Division of General Medical Sciences, Department of Medicine, Washington University School of Medicine, St Louis, Missouri; 4 Lawrence Memorial Hospital, New London, Connecticut

## Abstract

**Objective:**

To evaluate the attitudes of infectious diseases (ID) and critical care physicians toward antimicrobial stewardship in the intensive care unit (ICU).

**Design:**

Anonymous, cross-sectional, web-based surveys.

**Setting:**

Surveys were completed in March–November 2017, and data were analyzed from December 2017 to December 2019.

**Participants:**

ID and critical care fellows and attending physicians.

**Methods:**

We included 10 demographic and 17 newly developed, 5-point, Likert-scaled items measuring attitudes toward ICU antimicrobial stewardship and transdisciplinary collaboration. Exploratory principal components analysis (PCA) was used for data reduction. Multivariable linear regression models explored demographic and attitudinal variables.

**Results:**

Of 372 respondents, 315 physicians had complete data (72% attendings, 28% fellows; 63% ID specialists, and 37% critical care specialists). Our PCA yielded a 3-item factor measuring which specialty should assume ICU antimicrobial stewardship (Cronbach standardized α = 0.71; higher scores indicate that ID physicians should be stewards), and a 4-item factor measuring value of ICU transdisciplinary collaborations (α = 0.62; higher scores indicate higher value). In regression models, ID physicians (vs critical care physicians), placed higher value on ICU collaborations and expressed discomfort with uncertain diagnoses. These factors were independently associated with stronger agreement that ID physicians should be ICU antimicrobial stewards. The following factors were independently associated with higher value of transdisciplinary collaboration: female sex, less discomfort with uncertain diagnoses, and stronger agreement with ID physicians as ICU antimicrobial stewards.

**Conclusions:**

ID and critical care physicians endorsed their own group for antimicrobial stewardship, but both groups placed high value on ICU transdisciplinary collaborations. Physicians who were more uncomfortable with uncertain diagnoses reported preference for ID physicians to coordinate ICU antimicrobial stewardship; however, physicians who were less uncomfortable with uncertain diagnoses placed greater value on ICU collaborations.

In an era of progressive antimicrobial resistance, intensive care units (ICUs) represent incubators for the most drug-resistant pathogens and are the most frequent users of broad-spectrum antimicrobials. Nevertheless, the most recent antimicrobial stewardship guidelines do not make specific recommendations for antimicrobial stewardship in ICUs.^1^ In addition, the Society for Critical Care Medicine sepsis guidelines have not been endorsed by the Infectious Diseases Society of America (IDSA).^2^ Antimicrobial stewardship programs in ICUs have resulted in reductions in drug-resistant pathogen infections, broad-spectrum antimicrobial use, and antimicrobial costs, all without increases in mortality.^3,4^ Moreover, ID fellowship programs, full-time ID physicians, and clinical pharmacists with ID training have all been shown to reduce antimicrobial use.^5^ Transdisciplinary collaboration between infectious diseases (ID) and critical care practitioners has resulted in higher rates of guideline-adherent therapy and reductions in broad-spectrum antimicrobial use, mechanical ventilation, length of stay, hospital mortality, and healthcare costs.^6^ Much work remains to be done; studies have indicated that 30%–80% of antimicrobial use in ICUs is flawed by either being unnecessary or suboptimally prescribed.^7–11^

To be successfully implemented, an antimicrobial stewardship program in the ICU will need the buy-in of the treating physicians. Unfortunately, behavioral and social barriers to collaboration between ID and critical care physicians remain at many institutions. Therefore, our goal was to develop an online survey to assess the attitudes of ID and critical care physicians toward antimicrobial stewardship for sepsis and collaboration in the ICU.

## Methods

### Study population

Fellows and attending physicians in ID, pulmonary, and critical care medicine were eligible to participate in our anonymous, cross-sectional, web-based survey. Participants were recruited by e-mail using (1) listservs from the IDSA IDea Exchange Digest and the Society for Healthcare Epidemiology of America (SHEA) Open Forum Digest; (2) e-mails to program directors to disseminate the invitations to their trainees as part of their of ID, pulmonary, and critical care fellowship programs in the United States; and (3) e-mails to physicians in the ID, pulmonary, and critical care divisions at each study site, Washington University School of Medicine (WUSM) in St Louis and the University of New Mexico (UNM) School of Medicine in Albuquerque. Approval from the institutional review board was obtained at each study site. Potential participants were provided with information about the study after clicking the link in their e-mail, and informed consent was implied by survey completion.

### Research procedures

Between March and November 2017, invitations to participate were distributed 3 times over 2 months to eligible fellows and attending physicians via IDSA and SHEA listservs and WUSM and UNM institutional e-mails. In addition, as requested, fellowship directors in each specialty were e-mailed twice to ask them to disseminate study invitations to their trainees by e-mail.

### Survey

The 27-item web-based survey was developed by the study investigators and was pilot tested by 5 internal medicine physicians prior to study enrollment. These physicians were not included in the study sample, and pilot data were not included in the analyses for the larger study. Surveys were administered using Qualtrics Software (Qualtrics, Provo, UT). The first 10 questions collected demographic information regarding a respondent’s level of training (fellow or attending physician), subspecialty (critical care, ID, or critical care plus ID dual board certification), sex, and year of medical school graduation. We also asked about the type of hospital in which each physician primarily practices (academic or community-based), hospital size (0–250 beds, 251–500 beds, or ≥500 beds), primary practice location (within or outside the United States), average number of patients seen in the ICU each month (0–20 patients, 21–40 patients, 41–60 patients, or >60 patients), and whether their primary institution offered an antimicrobial utilization control (antimicrobial stewardship) program (yes or no). The remaining 17 items assessed attitudes regarding who should coordinate antimicrobial stewardship in the ICU, decisions about the use of antimicrobials for sepsis in the ICU, the value of transdisciplinary collaborations in the ICU, and the respondent’s discomfort with uncertain diagnoses. Responses to the attitudinal items used a 5-point Likert scale ranging from strongly disagree (1) to strongly agree (5).

### Statistical analysis

We used an iterative process of exploratory principal components analysis (PCA) with varimax rotation for data reduction of the newly developed attitudinal items. We did not rely solely on eigenvalues >1.000 to determine the number of components to retain for analysis; rather, we used the Lautenschlager parallel analysis criteria,^12^ based on the work of Velicer.^13^ We used Lautenschlager tables based on partial correlation matrices, which considered the number of items tested and the sample size to determine the minimum eigenvalue needed to retain a given number of factors. We retained items with high factor loadings ≥0.600 and eliminated items with factor loadings ≥0.400 on >1 factor. The internal consistency reliability of items on a factor was assessed using the Cronbach standardized α coefficient;^14,15^ items were further eliminated if α could be increased by eliminating those items. Although multiple-item scales with low α values might be considered as interpretable measures,^14^ we did not consider factors for additional analysis if the internal consistency reliability (α) for items on a factor were <0.60.^15^

We computed mean scores for newly developed multiple-item factors (after reverse coding items, as needed). We report descriptive statistics and results of 1-way analyses of variance examining between-group differences in mean (SD) attitudinal scores and mean number of years since medical school graduation, χ^2^ tests of association among various categorical demographic variables of interest, and Pearson correlations among continuous variables. Multivariable linear regression models identified variables that were independently associated with the newly developed attitudinal variables. All statistics were conducted using SPSS version 24.0 software (IBM, Armonk, NY). Two-tailed *P* values <.05 were considered significant.

## Results

Of 372 individuals who clicked on the survey link and responded to at least 1 item, we excluded 6 people who responded that they did not see any patients, on average, in the ICU each month and 8 people who were board certified in both ID and critical care due to their small numbers.

Before running the PCA, we eliminated 4 items (1, 2, 3, and 13) from further consideration because there was little variance in responses (ie, > 90% of respondents reported either strongly disagree/disagree or agree/strongly agree). The PCA thus began with 13 items and a sample of 340 respondents. We subsequently eliminated items 5 and 7, which did not yield >.600 for any factor. Also, 4 factors emerged from the PCA (Supplementary Table 1 online shows the survey items loading on each factor). The first 3-item factor that emerged measured physician attitudes toward which specialty should coordinate ICU antimicrobial stewardship; higher scores indicated greater agreement that ID physicians should be antimicrobial stewards (Cronbach standardized α = .71). The second, 4-item factor measured the value of ICU transdisciplinary collaborations; higher scores indicated greater value of collaboration (Cronbach standardized α = .62). Also, two 2-item factors emerged. For these factors, higher scores indicated greater agreement with situations in which they would use narrow-spectrum antimicrobials in the ICU before cultures are finalized (Cronbach standardized α = .48), and greater risk of poor outcomes when choosing narrow-spectrum antimicrobials in the ICU (standardized α = .44). Generally, an α ≥ .70 is desirable. Although “low intercorrelations can yield an interpretable scale,”^14^ these 2 factors with an α value <.60 were not analyzed further.

**Table 1.** Demographic Characteristics of the Sample (N = 315)

Characteristics	Participants (N=315)	Critical Care (N=116)	Infectious Disease (N=199)	*P* Value^a^
No. of years since completing medical school, mean (SD)^b^	17.7 (12.2)	11.0 (9.1)	21.6 (12.0)	<.001
**Level of training, no. (%)**				<.001
Fellow	87 (27.6)	65 (56.0)	22 (11.1)	
Attending	227 (72.4)	51 (44.0)	177 (88.9)	
**Sex, no. (%)**				.70
Female	132 (41.9)	47 (40.5)	85 (42.7)	
Male	183 (58.1)	69 (59.5)	114 (57.3)	
**Primary practice location, no. (%)**				.02
United States	289 (91.7)	106 (91.4)	183 (92.0)	
Canada	12 (3.8)	9 (7.8)	3 (1.5)	
Latin America	4 (1.3)	1 (0.9)	3 (1.5)	
Asia	8 (2.5)	0 (0.0)	8 (4.0)	
Europe	1 (0.3)	0 (0.0)	1 (0.5)	
Australia	1 (0.3)	0 (0.0)	1 (0.5)	
**Institution type, no. (%)**				<.001
Academic	244 (77.5)	112 (96.6)	132 (66.3)	
Community-based	71 (22.5)	4 (3.4)	67 (33.7)	
**Institution size, no. (%)**				<.001
0–250 beds	41 (13.0)	4 (3.4)	37 (18.6)	
251–500 beds	95 (30.2)	35 (30.2)	60 (30.2)	
>501 beds	179 (56.8)	77 (66.4)	102 (51.3)	
**Active antimicrobial stewardship program, no. (%)** ^c^				.047
Yes	301 (95.6)	107 (93.0)	194 (97.5)	
No	10 (3.2)	5 (4.3)	5 (2.5)	
Do not know	3 (1.0)	3 (2.6)	0 (0.0)	
**ICU patients seen per month, no. (%)**				<.001
0–20 patients	127 (40.3)	9 (7.8)	118 (59.3)	
21–40 patients	75 (23.8)	19 (16.4)	56 (28.1)	
41–60 patients	36 (11.4)	24 (20.7)	12 (6.0)	
≥61 patients	67 (21.3)	55 (47.4)	12 (6.0)	
Do not know	10 (3.2)	9 (7.8)	1 (0.5)	

Note. ICU, intensive care unit. SD, standard deviation.

a Tests of significance were 1-way analysis of variance (ANOVA) for number of years since medical school graduation and χ^2^ tests for all other comparisons between critical care and infectious diseases specialties.

b Five participants (3 in critical care and 2 in infectious disease) did not provide their year of graduation from medical school and were not included in the ANOVA.

c One participant in critical care did not respond to whether or not their primary institution had an antibiotic stewardship program and was not included in the χ^2^ test.

Our final sample included 315 individuals with complete data for all variables included in the regression analysis. Demographics of the 315 respondents included in the final sample (85% of 372 respondents) are shown in Table 1. Most participants were attending physicians (72.4%) and ID specialists (63.2%). Significant differences between ID and critical care physicians were observed in association with some demographic variables of interest but not sex. For example, a greater proportion of ID (vs critical care) physicians were attending physicians (88.9% vs 44.0%; *P* < .001). Of 315 physicians who responded to the survey, 289 (91.7%) were practicing in the United States and 95.6% reported that their institution had an active antimicrobial stewardship program.

We examined the differences by physician subspecialty in mean responses to each of the 17 attitudinal items and newly developed factors that emerged from the PCA (Table 2). ID physicians reported greater agreement with having “discomfort with uncertain diagnoses*”* (*P* = .03) and that they should “coordinate antibiotic stewardship*”* in the ICU (*P* < .001). critical care physicians reported greater agreement with the item, “Critical care physicians are the ones who should determine when and which antimicrobials to administer to most critically ill patients,” and the item, “In the ICU, solely the primary inpatient team understands the complexity of the case” (each *P* < .001).

**Table 2.** One-way Analysis of Variance of Means (SD) for Each Item Administered in the Survey by Physician Specialty Choice (N = 315)^a^

Item	Critical Care (N=116)	Infectious Disease (N=199)	*P* Value^a^
1. Critically ill patients with signs of sepsis should be treated empirically for most likely sources/pathogens, immediately after drawing blood and fluid cultures	4.7 (0.5)	4.7 (0.5)	.39
2. ill patients with signs of septic shock should be treated empirically for most likely sources/pathogens, immediately after drawing blood and fluid cultures	4.9 (0.4)	4.9 (0.3)	.25
3. If cultures are delayed by >2 hours, antibiotics should be administered as soon as possible in patients with sepsis	4.8 (0.6)	4.4 (0.7)	<.001
4. It is too risky to choose an empiric narrow spectrum antibiotic when treating patients in the ICU	3.6 (1.1)	3.3 (1.2)	.01
5. I am highly uncomfortable with uncertain diagnoses	2.4 (0.9)	2.7 (1.0)	.03
6. I would narrow antibiotics based on rapid diagnostic testing that is positive for influenza before cultures are finalized	2.9 (1.0)	3.5 (1.0)	<.001
7. I would narrow antibiotics at 48–72 hours in septic patients with negative cultures if clinically improving	4.5 (0.7)	4.4 (0.7)	.32
8. I would narrow antibiotics based on blood-culture gram stain before cultures are finalized	3.1 (1.2)	3.3 (1.2)	.14
9. Antibiotic resistance is the lesser of 2 evils when compared to early, broad-spectrum, empiric antimicrobial therapy for sepsis in critically ill patients	3.6 (0.9)	3.5 (1.1)	.14
10. Critical care physicians should determine when and which antimicrobials to administer to most critically ill patients	4.1 (0.7)	3.1 (0.9)	<.001
11. Infectious disease physicians should determine when and which antimicrobials to administer to most critically ill patients	2.6 (1.1)	3.7 (0.90)	<.001
12. In the intensive care units, antibiotic stewardship should be coordinated by infectious disease physicians	3.3 (1.1)	4.4 (0.7)	<.001
13. In general, clinical collaborations between the primary inpatient team and consultants would improve patient care in the ICU	4.3 (0.8)	4.8 (0.4)	<.001
14. In general, clinical collaborations are difficult in a stressful environment like the ICU	2.3 (1.1)	2.6 (1.2)	.02
15. In the ICU, solely the primary inpatient team understands the complexity of the case	2.3 (1.0)	1.8 (0.8)	<.001
16. Clinical collaborations take up too much time to be of significant value	1.7 (0.8)	1.6 (0.8)	.41
17. I strongly value transdisciplinary clinical collaborations in the ICU	4.6 (0.5)	4.6 (0.6)	.30

Note. ICU, intensive care unit; SD, standard deviation.

a Each item was coded using a 1–5-point scale; higher scores indicate greater agreement with each statement.

We examined the bivariate associations for each outcome of interest—preference that ID physicians should be the antimicrobial stewards in the ICU, and stronger endorsement of the value for transdisciplinary collaborations in the ICU—with each demographic variable (Table 3). Being an ID physician (vs critical care physician) and being an attending physician (vs fellow) were each associated with greater agreement with ID physicians coordinating antimicrobial stewardship in the ICU. Institution type and size and the number of patients seen in the ICU in a month were also associated with preference for ID physicians as antimicrobial stewards. Female physicians (vs male physicians) and physicians practicing in the United States (vs outside the United States) placed greater value on transdisciplinary collaborations in the ICU.

**Table 3.** One-Way Analyses of Variance of Each Newly Developed Factor, by Sample Demographic Variables (N = 315)

Characteristics	ID Physicians Should be Antimicrobial Stewards, Mean (SD) ^a^	*P* value	Value of ICU Transdisciplinary Collaborations, Mean (SD)^b^	*P* Value	Highly Uncomfortable With Uncertain Diagnoses, Mean (SD) ^c^	*P* Value
**Subspecialty**		<.001		.27		.03
Critical care	2.6 (0.7)		4.1 (0.6)		2.4 (0.9)	
Infectious diseases	3.7 (0.6)		4.2 (0.6)		2.7 (1.0)	
**Level of training**		<.001		.80		.93
Fellow	3.0 (0.8)		4.1 (0.6)		2.6 (0.8)	
Attending	3.4 (0.8)		4.1 (0.6)		2.6 (1.0)	
**Sex**		.33		.04		.12
Female	3.4 (0.8)		4.2 (0.6)		2.7 (0.9)	
Male	3.3 (0.9)		4.1 (0.6)		2.5 (1.0)	
**Primary practice location**		.89		.04		.001
United States	3.3 (0.9)		4.1 (0.6)		2.5 (1.0)	
Outside the United States	3.3 (0.7)		3.9 (0.7)		3.2 (1.2)	
**Institution type**		<.001		.33		.05
Academic	3.2 (0.8)		4.1 (0.6)		2.5 (1.0)	
Community-based	3.7 (0.7)		4.1 (0.6)		2.8 (1.1)	
**Institution size**		.01		.20		.07
0–250 beds	3.6 (0.6)		4.0 (0.6)		2.9 (0.9)	
251–500 beds	3.4 (0.8)		4.1 (0.7)		2.6 (1.00)	
>501 beds	3.2 (0.9)		4.1 (0.6)		2.5 (1.0)	
**Active antimicrobial stewardship program**		.53		.60		.08
Yes	3.3 (0.8)		4.1 (0.6)		2.6 (1.0)	
No	3.4 (1.1)		4.0 (0.7)		3.2 (0.9)	
Do not know	2.8 (0.5)		4.3 (0.5)		2.0 (0.0)	
**Average no. of ICU patients seen per month**		<.001		.04		.28
0–20 patients	3.5 (0.7)		4.2 (0.6)		2.6 (1.0)	
21–40 patients	3.6 (0.7)		4.2 (0.5)		2.6 (1.0)	
41–60 patients	3.0 (0.9)		4.0 (0.5)		2.6 (1.0)	
>61 patients	2.7 (0.9)		4.0 (0.7)		2.4 (1.0)	
Do not know	3.0 (0.8)		4.1 (0.4)		3.0 (0.94)	

Note. ICU, intensive care unit. SD, standard deviation. Tests of significance were one-way analysis of variance.

a Higher scores on the 3-item measure indicate greater agreement that ID physicians should be antimicrobial stewards in the ICU.

b Higher scores on the 4-item measure indicate greater value of transdisciplinary collaboration in the ICU.

c Higher scores on the single-item measure indicate greater discomfort with uncertain diagnoses.

Because we were interested in exploring whether one’s confidence might affect attitudes to collaboration between ID and critical care physicians, we included the item, “I am highly uncomfortable with uncertain diagnoses,” as a single-item measure (higher scores indicate being more uncomfortable) (Table 3). ID physicians (vs critical care physicians) reported being more uncomfortable with uncertain diagnoses. Additionally, being more uncomfortable with uncertain diagnoses was correlated with a longer time since graduation from medical school (*r* = .135; *P* = .02), greater preference for ID antimicrobial stewardship in the ICU (*r* = .152; *P* = .007), and less value placed on ICU transdisciplinary collaborations (*r* = −.213; *P* < .001). The 2 newly developed factors and the single-item measure of discomfort with uncertain diagnoses did not differ significantly by whether or not a physician’s institution had an active antimicrobial stewardship program. A greater number of years since graduation from medical school was significantly associated with greater agreement for ID antimicrobial stewardship in the ICU (*r* = .322; *P* < .001) and less value placed on ICU transdisciplinary collaborations (*r* = −.117; *P* = .039).

We ran multivariable linear regression models to identify variables that were independently associated with our 2 new multiple-item measures, preference for ID antimicrobial stewardship and greater value placed on transdisciplinary collaborations in the ICU, and the single-item measure of discomfort with uncertain diagnoses. In each model, we included the other 2 attitudinal measures as independent variables, in addition to the demographic variables of interest that were significantly associated with the new measures in bivariate tests, including institution size, physician subspecialty, physician training level, institution type, and sex. We observed a significant association between institution size and the number of patients that physicians saw in a month (*P* < .001); therefore, we included only institution size in multivariable models. Also, we did not include the physician’s primary practice location in the multivariable models because comparatively few physicians practiced outside the United States and a significantly greater proportion of female (vs male) physicians practiced in the United States versus outside the United States (43.6% vs 23.1%; *P* = .04).

In the first regression model (Table 4), greater preference for ID antimicrobial stewardship in the ICU was significantly associated with being an ID specialist, placing greater value on transdisciplinary collaborations, and reporting greater discomfort with uncertain diagnoses. In the second regression model (Table 5), greater value placed on transdisciplinary collaborations in the ICU was significantly associated with greater preference for ID antimicrobial stewardship in the ICU, female sex, and less uncomfortable with uncertain diagnoses. In the third regression model (Supplementary Table 2 online), being highly uncomfortable with uncertain diagnoses was associated with being an ID physician and with placing lower value for transdisciplinary collaborations in the ICU.

**Table 4.** Multivariable Regression Model to Identify Variables Independently Associated With the Preference for ID Physicians to Serve as Antibiotic Stewards in the ICU (N = 315)^a^

Variable	Standardized β	*P* Value
Value of transdisciplinary collaborations in ICU	0.151	.001
Highly uncomfortable with uncertain diagnoses	0.098	.03
Institution size	−0.054	.26
Physician subspecialty	0.604	<.001
Physician level of training	−0.079	.13
Institution type	0.055	.27
Sex	−0.007	.88

Note. ID, infectious diseases; ICU, intensive care unit.

a Categorical variables include institution size (1 = 0–250 beds; 2 = 251–500 beds; 3 ≥ 501 beds), physician subspecialty (1 = CC; 2 = ID), physician level of training (1 = fellow; 2 = attending), institution type (1 = academic; 2 = community based), and sex (1 = female; 2 = male).

**Table 5.** Multivariable Regression Model to Identify Variables Independently Associated With the Value of Transdisciplinary Collaboration in the ICU (N = 315)^a^

Variable	Standardized β	*P* Value
Prefer ID physicians as antibiotic stewards	0.230	.001
Highly uncomfortable with uncertain diagnoses	−0.240	<.001
Institution size	0.089	.13
Physician subspecialty	−0.034	.66
Physician level of training	0.026	.69
Institution type	−0.040	.52
Sex	−0.133	.02

Note. ICU, intensive care unit.

a Categorical variables include institution size (1 = 0–250 beds; 2 = 251–500 beds; 3 ≥ 501 beds), physician subspecialty (1 = CC; 2 = ID), physician level of training (1 = fellow; 2 = attending), institution type (1 = academic; 2 = community based), and sex (1 = female; 2 = male).

## Discussion

Our study has several interesting findings. First, critical care physicians were less likely to think they should be antimicrobial stewards of sepsis in the ICU than ID physicians. Secondly, ID physicians prefer that ID physicians be antimicrobial stewards, and they highly value transdisciplinary collaboration. Overall, ID and critical care physicians both favored their own group for antimicrobial stewardship, but both groups placed high value on transdisciplinary collaborations in the ICU. Years since completing medical school was a predictor of less value placed on transdisciplinary collaborations.

Our findings suggest that physicians uncomfortable with uncertain diagnoses prefer ID physicians as antimicrobial stewards in the ICU. On the other hand, physicians uncomfortable with uncertain diagnoses are less likely to value transdisciplinary collaboration, suggesting an element of fear, which has been shown to drive inappropriate antimicrobial use in other studies.^16^ Previous studies have shown that greater severity of sepsis increases mortality^17^ and that non-ICU patients do not experience worse outcomes with inappropriate initial antimicrobial therapy.^18^ Thus, lower sepsis severity commonly observed outside the ICU may partially explain differences in risk averseness between critical care physicians (who treat predominantly ICU patients) and ID physicians (who treat higher proportion of non-ICU patients), particularly because each hour delay of antibiotics for patients with septic shock increases mortality.^19^

Collaborative care has been shown to improve diagnosis and treatment of various health conditions.^20–23^ A recent workshop recommended that critical care physicians have antimicrobial stewardship as a core competency.^24^ However, the recommendation of this group was to incorporate antimicrobial stewardship objectives into critical care training, a daunting task in an already grueling fellowship. An alternative to increasing the number of competencies for critical care physicians would be to make interdisciplinary teams, including members who already have this training as part of their certification, such as ID physicians and pharmacists.

Human factors engineering and behavioral modification approaches are strategies that could be explored to rectify healthcare situations in which interpersonal or systemic factors preclude optimal care. Fear is often a barrier to appropriate antimicrobial use^16^ as are cultural norms and “prescribing etiquette.”^25^ Critical care physicians must be part of the solution; daily review of antimicrobials in the ICU improved patient outcomes.^26^ The solutions may vary by locale as well, considering that intensivists in Canada are supportive of antimicrobial stewardship programs, with only 11% finding this interaction an ineffective use of their time.^27^ The potential for improvements in patient care are significant—ID and ICU combined rounds reduce antimicrobial costs, ICU length of stay, hospital length of stay, and mortality.^28^

Our study has several limitations. We were unable to estimate the denominator for determining our participation rate because we did not know how many physicians actually received the invitation to participate. Therefore, our sample may not be representative of all ID and critical care specialists in the potential pool across the United States from which we drew our sample. However, the number of respondents exceeded 300, with sufficient power to detect relatively small differences between groups. Another possible limitation is the quantitative nature of our survey, which did not allow for discovery of particular human and systems factors that could impede or facilitate ICU antimicrobial stewardship and transdisciplinary collaborations. Future studies may benefit from mixed-methods approaches to explore in greater depth the various barriers to and facilitators of ICU antimicrobial stewardship and transdisciplinary collaboration. Nevertheless, the findings of our study are important. Antimicrobial stewardship in the ICU is associated with a variety of clinical benefits for patients and does not increase mortality.^29^ In addition, ICUs are breeding grounds for multidrug-resistant organisms, for which ID physicians can play a significant role in mitigating, thereby reducing risk of mortality.^30^ Recognition of the willingness of critical care physicians to implement ID-led antimicrobial stewardship in the ICU may increase the decision-making security of ID physicians and facilitate open communication and collaboration, which is a cornerstone of caring for critically ill patients. Results from this study may enable physicians to recognize their own biases, potentially making them more willing to accept collaborations in the ICU. Our long-term goal is to work toward a more unified and collaborative patient-care model in which patient outcomes are the central focus.

## Supplementary material

For supplementary material accompanying this paper visit https://doi.org/10.1017/ice.2021.389. click here to view supplementary material
