# Supplementary material for: Antimicrobial stewardship for sepsis in the intensive care unit: Survey of critical care and infectious diseases physicians
Source: Infect Control Hosp Epidemiol. 2022 Aug 12;43(10):1368–74. doi: 10.1017/ice.2021.389 (PMC9588438; doi:10.1017/ice.2021.389)
Supplement: Supplementary file 1 [file S0899823X21003895sup.zip › S0899823X21003895sup001.docx]

**Appendix 1: Survey instrument**

1. Current level of training:

- fellow
- attending physician
- other (survey ends here)

2. Subspecialty:

- critical care
- infectious diseases
- critical care/infectious disease dual-boarded

3. Sex:

- female
- male

4. Year of medical school graduation:

(fill in the blank)

5. Geographic location in which you primarily practice:

- United States
- Canada
- Latin America
- Asia
- Europe
- Australia

6. If you chose critical care, please specify type of ICU (select all that apply):

- MICU (medical intensive care unit)
- SICU (surgical including trauma intensive care unit)
- CTICU (cardio-thoracic intensive care unit)
- NICU (neurology including neurosurgical intensive care unit)
- PICU (pediatric including neonatology intensive care unit)

7. Your primary institution is:

- academic
- community-based

8. Size of your primary institution:

- 0-250 beds
- 251- 500 beds
- 501-1000 beds
- >1000 beds
- Do not know

9. Is there an antibiotic utilization control program (antibiotic stewardship) at your primary institution?

- Yes
- No
- Do not know

10. On average, how many patients do you see in the ICU each month??

- 0
- 1 – 20
- 21 – 40
- 41 – 60
- 61 – 80
- 81 – 100
- >100
- Do not know

For the next set of questions, we use the following definitions:

***Sepsis*** *is defined as organ dysfunction due to a dysregulated host response to infection*

***Septic shock*** *is defined as hypotension requiring vasopressor support to maintain a mean arterial pressure ≥65 mmHg and a lactate level >2 mmol/L despite adequate fluid resuscitation (approx 3L).*

Keeping these definitions in mind, please rate your level of agreement with each of the following statements.

|  | Strongly agree | Agree | Neither agree nor disagree | Disagree | Strongly disagree |
| --- | --- | --- | --- | --- | --- |
| 1. Critically ill patients with signs of **sepsis** should be treated empirically for most likely sources/pathogens, immediately after drawing blood and fluid cultures | □ | □ | □ | □ | □ |
| 2. Critically ill patients with signs of **septic shock** should be treated empirically for most likely sources/pathogens, immediately after drawing blood and fluid cultures | □ | □ | □ | □ | □ |
| 3. If cultures are delayed by more than two hours, antibiotics should be administered as soon as possible in patients with **sepsis** | □ | □ | □ | □ | □ |
| 4. It is too risky to choose an empiric narrow spectrum antibiotic when treating patients in the ICU | □ | □ | □ | □ | □ |
| 5. I am highly uncomfortable with uncertain diagnoses | □ | □ | □ | □ | □ |
| 6. I would narrow antibiotics based on rapid diagnostic testing that is positive for influenza before cultures are finalized | □ | □ | □ | □ | □ |
| 7. I would narrow antibiotics at 48-72 hours in septic patients with negative cultures if clinically improving | □ | □ | □ | □ | □ |
| 8. I would narrow antibiotics based on blood culture Gram stain before cultures are finalized | □ | □ | □ | □ | □ |
| 9. Antibiotic resistance is the lesser of two evils when compared to early, broad-spectrum, empiric antimicrobial therapy for sepsis in critically ill patients | □ | □ | □ | □ | □ |
| 10. Critical care physicians should determine when and which antimicrobials to administer to most critically ill patients | □ | □ | □ | □ | □ |
| 11. Infectious disease physicians should determine when and which antimicrobials to administer to most critically ill patients | □ | □ | □ | □ | □ |
| 12. In the intensive care units, antibiotic stewardship should be coordinated by infectious disease physicians | □ | □ | □ | □ | □ |

| 13. In general, clinical collaborations between the primary inpatient team and consultants would improve patient care in the ICU. | □ | □ | □ | □ | □ |
| --- | --- | --- | --- | --- | --- |
| 14. In general, clinical collaborations are difficult in a stressful environment like the ICU. | □ | □ | □ | □ | □ |
| 15. In the ICU, solely the primary inpatient team understands the complexity of the case. | □ | □ | □ | □ | □ |
| 16. Clinical collaborations take up too much time to be of significant value. | □ | □ | □ | □ | □ |
| 17. I strongly value transdisciplinary clinical collaborations in the ICU. | □ | □ | □ | □ | □ |
