# Supplementary material for: Antimicrobial stewardship for sepsis in the intensive care unit: Survey of critical care and infectious diseases physicians
Source: Infect Control Hosp Epidemiol. 2022 Aug 12;43(10):1368–74. doi: 10.1017/ice.2021.389 (PMC9588438; doi:10.1017/ice.2021.389)
Supplement: Supplementary file 1 [file S0899823X21003895sup.zip › S0899823X21003895sup002.docx]

**Supplemental Table 1.** Factor Loadings from Principal Components Analysis Related to Antimicrobial Stewardship, Usage, and Clinical Collaboration in the ICU (N = 340)

|  | **Factor 1** Cronbach’s Alpha = .71 | **Factor 2** Cronbach’s Alpha = .62 | **Factor 3** Cronbach’s Alpha = .44 | **Factor 4** Cronbach’s Alpha = .48 |
| --- | --- | --- | --- | --- |
| **ID physicians should coordinate antibiotic stewardship in the ICU** |  |  |  |  |
| Item 10: “Critical care physicians should determine when and which antimicrobials to administer to most critically ill patients”^a^ | **-.767** | .020 | .166 | -.013 |
| Item 11: “ID physicians should determine when and which antimicrobials to administer to most critically ill patients” | **.787** | -.047 | .080 | .153 |
| Item 12: “In the ICU, antibiotic stewardship should be coordinated by ID physicians” | **.782** | -.033 | -.022 | .055 |
| **Valuing clinical collaborations in the ICU** |  |  |  |  |
| Item 14: “In general, clinical collaborations are difficult in a stressful environment like the ICU”^b^ | .131 | **.670** | .099 | .066 |
| Item 15: “In the ICU, solely the primary inpatient team understands the complexity of the case”^b^ | -.376 | **.556** | .288 | .054 |
| Item 16: “Clinical collaborations take up too much time to be of significant value”^b^ | -.097 | **.803** | .027 | .111 |
| Item 17: “I strongly value transdisciplinary clinical collaborations in the ICU” | .045 | **-.660** | .174 | .220 |
| **Using narrow-spectrum antibiotics is too risky in the ICU** |  |  |  |  |
| Item 4: “It is too risky to choose an empiric narrow spectrum antibiotic when treating patients in the ICU” | -.041 | .132 | **.778** | -.050 |
| Item 9: “Antibiotic resistance is the lesser of two evils when compared to early, broad-spectrum, empiric antimicrobial therapy for sepsis in critically ill patients” | -.047 | -.063 | **.753** | -.113 |
| **Situations in which one would narrow antibiotics in the ICU before cultures are finalized** |  |  |  |  |
| Item 6: “I would narrow antibiotics based on rapid diagnostic testing that is positive for influenza before cultures are finalized” | .288 | .058 | -.062 | **.724** |
| Item 8: “I would narrow antibiotics based on blood culture Gram stain before cultures are finalized” | -.059 | -.025 | -.115 | **.832** |

Abbreviations. *ICU*: Intensive Care Unit; *ID*: Infectious Diseases.

Note: Response options ranged from (1) Strongly Disagree to (5) Strongly Agree. Items loading on a factor are shown bold.

^a^ Item subsequently reverse coded to compute the new multi-item variable for analysis, so that higher scores indicate greater agreement that ID physicians should coordinate antibiotic stewardship in the ICU.

^b^ Item subsequently reverse coded to compute the new multi-item variable for analysis, so that higher scores indicate greater agreement with the value of clinical collaborations in the ICU.

**Supplemental Table 2. Multivariable regression model to identify variables independently associated with feeling highly uncomfortable with uncertain diagnoses (N=315)^a^**

|  | **Standardized Beta** | ***P* value** |
| --- | --- | --- |
| Prefer ID physicians as antibiotic stewards | 0.151 | .03 |
| Value of clinical collaborations in ICU | -0.244 | < .001 |
| Institution size | -0.048 | .43 |
| Physician subspecialty | 0.043 | .58 |
| Physician level of training | -0.061 | .34 |
| Institution type | 0.052 | .41 |
| Sex | -0.101 | .07 |

^a^Categorical variables include Institution size (1= 0-250 beds, 2= 251-500 beds, 3 ≥ 501 beds), Physician subspecialty (1= CC, 2= ID), Physician level of training (1= Fellow, 2= Attending), Institution type (1= Academic, 2= Community-based), and Sex (1= Female, 2= Male)
